# Drugs to affect the smooth musculature of the human ureter - an update with integrated information from basic science to the use in medical expulsion therapy (MET)

**DOI:** 10.1007/s00345-024-05368-5

**Published:** 2024-11-28

**Authors:** Petter Hedlund, Harrina E. Rahardjo, Dimitrios Tsikas, Markus A. Kuczyk, Stefan Ückert

**Affiliations:** 1 Faculty of Medicine, Department of Clinical Pharmacology, Linköping University, Linköping, Sweden; 2 School of Medicine, Department of Urology, University of Indonesia, Cipto Mangunkusumo Hospital, Jakarta, Indonesia; 3 Hannover Medical School, Centre of Pharmacology & Toxicology, Core Unit Proteomics, Hannover, Germany; 4 Hannover Medical School, Division of Surgery, Department of Urology & Urological Oncology, Hannover, Germany

**Keywords:** Ureter, Pharmacology, Upper urinary tract stone disease, Treatment options

## Abstract

**Purpose:**

Urolithiasis and symptomatic ureterolithiasis represent diseases known to be on the increase in most westernized countries. The present article aims to give an overview on some drug principles assumed to target signalling systems involved in modulating ureter smooth muscle contractility and to present background to their potential use or prospects in ureter stone disease.

**Methods:**

The article reviews drugs that have been evaluated over the last decades in vitro, in vivo and/or in clinical settings with regard to their properties to achieve spontaneous passage of (distal) ureteral stones and relieve colic pain. Among these drugs are alpha- and beta-adrenoceptor antagonists, calcium channel blocking agents, Rho kinase inhibitors, nitric oxide (NO) donor drugs, selective inhibitors of cyclic nucleotide phosphodiesterase enzymes (PDEs), as well as potassium channel openers.

**Results:**

Based on the recent scientific information on agents targeting different pathways, antagonists of alpha 1-adrenoceptors, inhibitors of the PDE isoenzymes PDE4 and PDE5 (affecting cyclic AMP- or NO/cyclic GMP-mediated signals that facilitate relaxation of ureter smooth muscle), as well as the combination of certain drugs (for example, PDE5/PDE4 inhibitor plus alpha 1-AR antagonist) seem to be intriguing pharmacological approaches to medical expulsion therapy (MET) in the overall population of patients.

**Conclusion:**

While NO donors, calcium channel antagonists and potassium channel openers may be limited for further development for medical expulsion therapy (MET) due to their systemic effects and a lack of effect on stone clearance, Rho kinase inhibitors should be explored further as a future pharmacological principle in ureteral stone disease.

## Introduction

The ureters are tubes composed of smooth muscle that transport urine from the kidneys to the urinary bladder. They are lined with urothelial cells, a form of transitional epithelium, and feature an extra layer of smooth muscle in the lower third to aid in peristalsis. The main function of the human ureter is to transport urine containing metabolic debris from the kidney to the urinary bladder. This essential action is achieved by unidirectional peristalsis that also protects the upper urinary tract from backflow and increased pressures that could otherwise damage the kidney parenchyma and also the vital functions of the nephron. Peristalsis is triggered in the renal pelvis by spontaneously electrical bursts in so-called pacemaker cells, i.e. interstitial Cajal-like cells and atypical smooth muscle cells [1]. Intercellular coupling via gap junctions forms a basis for excitation-contraction that synchronizes the propulsive activity from the pyelo-ureteric junction to and along the ureter [2, 3]. The autonomous peristaltic contractile activity of the ureters can be modulated by intrinsic signals from nerves, the urothelium and interstitial cells, and may also be affected by inflammatory or infectious processes [4, 5]. Alterations of normal peristaltic function of the ureter are related to clinical manifestations, such as ureter stone disease, vesico-ureteric reflux and upper urinary tract infections [1, 6]. Stone disease is a common condition worldwide and global trends have been reported of increasing occurrence of upper urinary tract stones. In addition, 5-year recurrence of symptomatic stone episodes after clinical care are seen in roughly half of these patients [7, 8]. Obstruction of the ureter pathways in stone disease has immediate impact on the transport of urine and causes proximal build-up of pressure that may present as severe episodes of renal colic in these patients. Depending on stone characteristics, the current clinical management includes pain control, surgical extraction, extracorporeal shock wave therapy (ESWL) and medical expulsion therapy (MET) directed towards regulatory sites of ureteral smooth musculature, as well as therapies that target metabolic aspects of stone formation [9, 10]. While research into transmitters, receptors and intracellular mediators in lower urinary tract function and dysfunction has resulted in the clinical implementation of several pharmacological principles for the treatment of symptoms, signalling functions in ureteral physiology and pathophysiology require further clarification in order to recommend distinct pharmacological interventions in ureter stone disease [6]. Some drugs that have been identified and evaluated for MET are available as an optional therapy prior to stone extraction or in the case that surgical intervention is not recommended. Theoretically, in order to dislodge a stone and allow it to pass, any drug for MET should not only have an effect on the autonomous peristaltic contractions but also reduce basal tonus or local spasms of the ureter. The most frequently used agents are alpha-adrenoceptor (alpha-AR) antagonists [11, 12]. However, it is suggested that there still is a need for additional large scale clinical studies to gain definite evidence of the clinical benefit of this therapeutic approach in stone disease [10, 13]. In addition, there is also a need to identify novel pharmacological targets that are involved in the regulation of ureteral peristalsis and to assess their potential for improving passage of upper urinary tract calculi.

The current paper aims to give an overview on some drug principles assumed to target signalling systems involved in modulating ureter smooth muscle contractility and to present background to their potential use or prospects in ureter stone disease.

## Alpha 1-adrenoceptor (AR) antagonists

Sympathetic adrenergic signals belong to the most extensively explored pathways involved in modifying the contractility of the ureter. Briefly, the ureters are densely supplied by adrenergic nerves and expresses large amounts of alpha-ARs, with alpha 1-ARs being more abundant than other adrenoceptors. Characterization of the expression of both genes and protein of alpha 1-AR subtypes in the proximal, medial and distal region of the human ureter disclosed the presence of all subtypes with the highest density in the distal ureter over the middle and proximal parts. Overall, the rank order of subtype expression displayed a predominance of the alpha 1D and alpha 1 A AR in the proximal, middle and distal sections of the ureter. The four alpha 1-AR subtypes are predominantly coupled with G-proteins of the Gq/11 family to activate the phospholipase C mechanism, that, in turn, induces smooth muscle contraction [14–16]. Isolated ureter tissue responds with contractile activity to alpha-AR agonists and the activation of alpha-ARs has positive chronotropic effects on ureter peristalsis and increases basal intraluminal pressure [1, 6]. Effects induced by alpha 1-AR antagonists on ureter contractility have been extensively investigated in isolated tissues as well as in vivo animal models. In these investigations, substantial evidence was presented for inhibitory effects by alpha 1-AR antagonists on the contractile activity of isolated tissue from several species, including humans [1, 17]. In studies examining subtype-selective agents, the alpha 1 A-AR has been reported to be of major importance for ureter contractions induced by agonists or activation of nerves [15–19]. It should be noted that characterization of subtype selectivity of some of these agents may be revisited depending on the method and species used [20]. For example, a recent comprehensive investigation of the selectivity profiles of several agents on human alpha 1-AR subtypes disclosed that naftopidil, previously considered to be partly selective to the alpha-1D receptors, exhibited better affinity to alpha 1 A [21]. In in vivo animal studies, alpha 1-AR antagonists have been described to reduce intraluminal pressures in both non-obstructed and obstructed ureters [17, 22–25]. However, the data on the effects of alpha 1-AR antagonists on inotropic and chronotropic peristaltic contractile activity (frequency or amplitude of contractions) also vary to a certain degree between the different investigations, depending on the species (rat, pig, dog), the methods used and the drug dosages applied. The findings of decreased basal ureteric pressures induced by alpha 1-AR antagonists are, in general, consistent with results obtained in humans [17, 22, 23, 26]. Taken together, information from bench-to-bedside on the role of alfa1-AR-mediated functions in modifying ureter contractility forms a basis for the use of alpha1-AR antagonists in MET in selected cases of ureter stone disease [10, 13]. Recent meta-analyses of trials empanelling more than 10.000 patients using alpha 1-AR antagonists such as alfuzosin, tamsulosin, doxazosin, naftopidil or silodosin, mostly for lower ureteric stones of less than 10 mm in diameter, revealed that alpha 1-AR antagonists improve stone clearance, reduce the use of analgetics and also the duration of hospitalization [26–28]. Secondary outcome reports on the impact the alpha 1-AR antagonists on stone expulsion rates varied between meta-analyses. Campschroer et al. (2018) did not find any difference between the drugs administered whereas Sharma et al. (2022) suggested that silodosin, followed by alfuzosin and tamsulosin, is most efficacious for MET of lower ureter stones. Reported off-target adverse events were related to the drug class and target receptors and included dizziness, hypotension and retrograde ejaculation. Major adverse events have been reported in only 5 out of 1000 patients, discontinuation due to treatment with alpha 1-AR antagonists was 0.6% versus 0.07% in the placebo group [26, 28, 29]. Even if alpha 1-AR antagonists have been well explored for MET, remarks on variability in efficacy between the studies and notations on low to moderate quality of evidence from a number of trials have raised concerns on the notion that alpha 1-AR antagonists are able to consistently promote stone expulsion [11, 12, 26, 30].

## Beta-adrenoceptor agonists

All three beta-AR subtypes have been identified in the ureter from various species including man at the levels of mRNA and protein [31]. Isolated ureter preparations from rats, pigs, rabbits, guinea-pigs, dogs and humans that were spontaneously active, activated by the addition of constrictor compounds or by the stimulation of nerves, responded with decreased contractility to beta-AR agonists. In particular, isolated human ureteral tissue was potently and selectively relaxed by KUL-7211, a beta 2/beta 3-AR agonist [32–34]. These effects are reported to be reversed by beta-AR antagonists [35–38]. However, inhibitory effects by the beta 3-AR agonist mirabegron on contractions of the pig ureter were recently challenged and suggested to be mediated by off-target effects via alpha 1-ARs [33]. In in vivo animal models of non-obstructed or obstructed ureters, beta-AR agonists reduced intra-ureteral pressures [39, 40]. Besides reducing tonic contractile activity of the ureter, these agents were also reported to reduce spontaneous activity in vitro and inotropic/chronotropic peristaltic contractile activity in vivo [25, 34, 35, 37]. Information on which beta-ARis are considered to be of utmost importance for the control of ureter contractility varies between the various studies and is likely related to species differences, diverse methodological approaches, as well as variabilities in the pharmacological tools used. Beta-AR agonists have previously been assessed as pharmacological approaches to reduce pain associated with renal colic secondary to upper urinary tract stones. A meta-analysis was unable to identify any eligible studies that meet the inclusion criteria for assessment, and concluded that there is insufficient evidence for the use of these agents to counteract events of renal colic [41]. Results from a study to assess whether salbutamol, a beta 2-AR agonist, could be used as an add-on drug treatment in ureteric colic were negative [42] (also see: www.isrctn.com/ISRCTN14552440). In a meta-analysis of seven studies consisting of 728 patients, the clinical potential of the beta 3-AR agonist mirabegron in MET has been evaluated for MET in ureteric stone disease in seven studies consisting of 728 patients. Albeit some concerns of risk of bias (mainly in the domain *Selection of Reported Results*) were raised for 6 out of the 7 studies, it was concluded that the agent increases stone expulsion rate and reduces frequency of pain episodes. No difference in efficacy could be verified on endpoints between mirabegron, tamsulosin and silodosin. The side-effects to mirabegron in the included studies were reported to be rare with two patients complaining of nausea and dry mouth and two patients with hypertension who thus discontinued treatment [43].

## Muscarinic receptor agonists/antagonists

The ureters are described to reach a very rich supply of cholinergic nerves and, as assessed by the presence of mRNA or protein, also express all five subtypes of muscarinic receptors [44]. However, data on the role of the activation of muscarinic receptors in isolated ureter preparations from animals and humans are sparse, not recent, and vary from no overt effects to increased contractile responses and inhibition of contractions [45–47]. Similarly, in vivo, in animal models, muscarinic receptor agonists are described to exhibit either no activity or to exert variable actions on ureter contractions and peristaltic frequency [48, 49]. To date, only two marginal studies have evaluated the potential of the muscarinic receptor antagonist tolterodine tartrate for ureter MET. No improvement in expulsions rates albeit possible positive effects on pain episodes were reported [50, 51]. In fact, given the cumulated scientific data available from the very few investigations conducted up until today, more research efforts utilizing elaborated in vitro models, as well as clinical studies, are strongly indicated in order to establish profound evidence supporting the role of the activation or blockade of muscarinic receptors in the treatment of upper urinary tract stones disease.

## Nitric oxide (NO) donor drugs

NO induces relaxation of vascular and non-vascular smooth muscle by activating the cytosolic enzyme soluble guanylyl cyclase (sGC), thereby increasing the tissue levels of the second messenger cGMP. This, in turn, interacts with various intracellular components regulating the activities of proteins involved in smooth muscle contraction [52]. Both nerves and the urothelium are proposed as endogenous sources of NO in the ureters. In the ureters from rats, pigs and humans, nerves containing NO synthase (NOS) have been demonstrated and found to be more frequent at distal sections and at the uretero-vesical junction [53–56]. Suggesting a functional inhibitory role of these nerves, pharmacological inhibition of the synthesis of NO during contractions induced by transmural activation of ureter preparations from humans increased contractile responses [57]. NOS has been located to ureter urothelial cells that have been shown to produce NO [58]. Exogenous NO and NO donors, such as sodium nitroprusside (SNP) and linsidomine (SIN-1), have been shown to counteract contractions of the human ureter induced by electrical field stimulation. Responses by SIN-1 were described to be mediated via cGMP-mediated mechanism as the pre-exposure of the tissue to methylene blue, an inhibitor of the cGMP-producing enzyme sGC, significantly reduced the relaxations. Similarly, Rp-8-pCPT-cGMPS, an inhibitor of the cyclic GMP-binding protein kinase G, counteracted SIN-1-mediated relaxant responses in human ureter preparations [54]. In accordance, responses to NO donors have also been shown to be accompanied by increased levels of cyclic GMP in ureter preparations exposed to these agents [55, 56]. Overall, these findings verify that relaxation of ureteral smooth muscle involves the NO/cyclic GMP pathway and indicate that this may hint to new therapeutic approaches to effectively treat colic events due to ureteral stones. However, this assertion is only in part supported by the clinical use of the NO donor drugs isosorbide dinitrate (ISDN) and glycerol trinitrate (GTN) to either relieve pain sensations caused by renal colic or prevent pain episodes triggered by calculi in the renal pelvis or ureter. Adding ISDN to tenoxicam, a non-steroidal anti-inflammatory drug with analgetic properties, in 50 patients presenting with symptoms of renal colic resulted in a significant decrease in the severity of pain sensations when compared to the administration of tenoxicam alone [59]. In a study that empaneled 50 consecutive patients who had clinically confirmed ureteral calculi of less than 10 mm in diameter, patients were randomized to receive for 6 weeks patches containing either GTN or placebo. The patients kept a diary to record pain episodes and were clinically reviewed once a week. In total numbers, those patients who had been randomized to the GTN patches experienced less pain episodes when compared to the placebo group. During the treatment period, approximately 30% of the patients in the GTN group discontinued treatment, mainly because of persisting headaches. In summary, despite a tendency in favour of the group that had received GTN, the rate of treatment success did not achieve any statistical significance, so that the authors were unable to declare any advantages in using GTN [60].

## Phosphodiesterase (PDE) inhibitors

Cyclic adenosine- and guanosine-3’,5’-monophosphate (cyclic AMP, cyclic GMP) are important intracellular second messengers that trigger various transduction cascades that also comprise activation of cyclic nucleotide-dependent protein kinases and subsequent phosphorylation of the actin-myosin system, as well as membrane-located Ca^2+^-channels involved in the regulation of smooth muscle tonus [61, 62]. Cyclic nucleotides are degraded by phosphodiesterase (PDE) isoenzymes, a heterogeneous group of hydrolytic enzymes. PDEs are mainly classified according to their preference for cyclic AMP or/and cyclic GMP, kinetic parameters of cyclic nucleotide hydrolysis, sensitivity to inhibition by various compounds, and allosteric regulation by other molecules. So far, eleven (11) families of PDE isoenzymes with additional splice-variants have been distinguished [63]. Since the distribution and functional significance of PDE isoenzymes vary significantly in different tissues, selective inhibition of the activity of PDE isoenzymes has the potential to exert specific effects on the target tissue. To date, 6 out of the 11 PDE isoenzymes have been proven to be of pharmacological significance in therapies for heart and pulmonary disease, sexual dysfunctions and lower urinary tract symptoms [63–71]. Taher et al. (1994) were the first to reveal the activities of the PDE isoenzymes 1, 2, 4 and 5 in cytosolic supernatants prepared from human ureteral tissue. In isolated ureters, they demonstrated the properties of quazinone (PDE3 inhibitor), rolipram (PDE4 inhibitor) and zaprinast (a dual inhibitor of the PDE5 and PDE1) to reverse the tonic contractions induced by KCl [72]. Later, Kühn et al. (2000) confirmed the relaxing properties of inhibitors of PDE4 (rolipram) and PDE5 (zaprinast, E 4021, morpholinosulfonyl-pyrazolopyrimidine) on isolated human ureteral smooth musculature. They also reported that these effects were paralleled by a several-fold elevation in intracellular levels of cyclic AMP (3- to more than 30-fold) and cyclic GMP (up to 2-fold) [73]. Becker et al. (1998) examined the potential of the PDE4 inhibitor rolipram in vivo, in the rabbit, in comparison to the non-specific PDE inhibitors papaverine and theophylline to exert relaxation of the ureter. In support of the in vitro data presented by Kühn et al. (2000), they found that rolipram significantly affected ureteral peristalsis (frequency, intra-ureteral pressure, amplitude of intraluminal pressure) with no considerable effects on the systemic circulation. Papaverine or theophylline that caused a significant depression in systemic blood pressure, had only short-lasting effects on ureter function [74]. Drotaverine hydrochloride, described as an inhibitor of the PDE4 “*structurally similar to papaverine*”, was later evaluated in 102 patients with colic due to renal or ureteric stones and was proven to be effective to reduce pain in 80% of patients compared to 46% in the placebo group. Expulsion of stones was not included as an endpoint in the study. The most frequent side effects noted in 20 out of 48 patients who received the PDE4 inhibitor were a transitory decrease in blood pressure, vertigo, nausea or vomiting [75]. Gratzke et al. (2007) in their experimental set-up investigated the effects of the selective PDE5 inhibitors sildenafil, tadalafil and vardenafil on the tension induced by KCl of isolated human ureteral smooth muscle and the turn-over of cyclic nucleotides in the tissue. Surprisingly, the production of cyclic GMP was elevated (3-fold) only in the presence of vardenafil while the exposure of the ureteral smooth muscle to sildenafil or tadalafil did not result in a marked increase in cyclic GMP levels. In contrast to the results presented by Kühn et al. (2000), no rise in cyclic AMP was induced by either drug [76]. In clinical evaluations for MET, a meta-analysis of 3 studies including 256 participants found that expulsion rates of ureteral calculi were significantly better with sildenafil and tadalafil in comparison to placebo. A sub-analysis of efficacy between tadalafil and tamsulosin yielded no difference between the two treatments [77]. In contrast, another meta-analysis from four studies including around 600 patients found that tadalafil exhibited better expulsion rates, a lower need for analgetics than tamsulosin, but had a higher rate of drug-related adverse events. Also, the combination of tamsulosin and tadalafil was assessed to be superior as MET over tamsulosin alone [78]. Comparing the efficacy of various drugs or drug combinations for MET of distal ureter stones in data from 50 randomized studies with more than 12.000 patients, Sharma et al. (2020) found that tadalafil along with alfuzosin, tamsulosin or silodosin were superior to placebo, whereas data for sildenafil did not achieve this level. Using the surface under the cumulative ranking curve method, the combinations tadalafil plus silodosin and tadalafil plus tamsulosin scored very well on stone expulsion rate and expulsion time [79].

## Rho kinase (ROCK) inhibitors

Rho-associated coiled kinases (ROCK) play an important role in the regulation of actin-myosin interaction and cytoskeletal function and, as such, serve as an important part in the control of smooth muscle tonus. Effects are related to Ca^2+^-independent changes of the interaction between actin and myosin via inhibition of myosin-light chain (MLC) phosphatase that results in contraction of smooth muscle. Two isoforms with similarities in the enzymatic region, ROCK 1 and ROCK 2, are expressed in animal and human tissues [80]. There is emerging interest in ROCK-mediated signals for the development of new pharmacological approaches to cardiovascular and pulmonary diseases, as well as to lower urinary tract dysfunctions [81–83]. For the upper urinary tract, the ROCK isoenzymes have been located also in ureteral tissue from humans [84]. Interestingly, it has been reported from experiments in rabbits that the expression of both ROCK 1 and ROCK 2 increases in obstructed ureters after ligation at the vesico-ureteric junction [85]. Upon transmural activation of nerves of the sheep ureter, the ROCK inhibitors Y 27632 and fasudil significantly inhibited contractions, and in isolated rat, rabbit and human ureters, Y 27,632 also counteracted electrically induced contractions, potentially by reducing MLC phosphorylation and also L-type Ca^2+^ entry. In the isolated rat, rabbit, sheep, pig and human ureters, ROCK inhibitors also counteracted agonist-induced contractions of ureteral smooth muscle preparations [85–88]. To the best of our knowledge, in vivo assessment of ROCK inhibition on ureter contractions has only been performed in rats. In a model for partial obstruction with simulated normal urine production, intravenous administration of Y 27632 had no effect on ureteric peristaltic frequency, but reduced tonic intra-ureteral pressures, as well as wave peristaltic amplitude pressure of the obstructed ureter. Minimal effects on the contralateral non-obstructed ureter were recorded [89]. In the pig, in the research for drugs administered locally to relax the ureter, both the Ca^2+^ antagonist nifedipine and Y 27632 were administered intra-luminally and found to be most effective in reducing the frequency and amplitude of ureteral contractions when compared to the untreated baseline [88]. Unfortunately, as the two agents were administered together, it cannot be assessed which effects relate to which compound. Up until today, drugs acting to inhibit the ROCK pathway have not been evaluated for MET, however, this pharmacological principle definitely merits further investigations in ureter stone disease.

## Calcium (Ca2+) channel antagonists

Calcium has a central role for excitation-contraction coupling and is a main regulator of ureteric smooth muscle contraction [6]. Voltage-sensitive Ca^2+^ currents have been recorded in electro-physiological experiments of ureteric smooth muscle cells and Ca^2+^ channel antagonists been described to inhibit spontaneous, tonic or nerve-induced contractions of isolated ureteric preparations from animals and humans [90–92]. In vivo, in pigs, the intraluminal application of verapamil caused a transient increase in peristaltic activity and induced dilatation of the proximal ureter for up to 1 h, however, the drug had no effect on the diameter of the middle or distal sections of the ureter [93]. Clinical trials on nifedipine, either as a stand-alone treatment or combined with corticosteroids, conducted in humans yielded variable, inconclusive effects on intraluminal ureteric pressure, peristalsis and expulsion rates in patients scheduled for ureteroscopy due to stone disease. The trials that looked into its effectiveness found that the drug is not superior to the alpha 1-AR antagonist tamsulosin but was linked to a much greater frequency of side effects, such as headaches, nausea and drowsiness [23]. Despite the lack of solid data in humans and scarce information from in vivo animal models, the drug principle has been tested further for MET. However, meta-analyses of clinical trials using nifedipine for ureter stones showed that the agent had either little or even no effect in promoting expulsion rates or pain episodes [27, 79].

## Potassium (K+) channel openers (PCOs)

It has been well established that the tension of smooth muscle is, in part, regulated by the activity of K^+^-channels located in the outer cell membrane. Four main families of K^+^ channels, each with multiple subfamilies, are widely distributed in the body and play key roles in regulating cellular excitability, influencing membrane resting potential, and responding to various stimuli like voltage changes and intracellular calcium levels. The opening of K^+^-channels leads to an increase in K^+^-conductance, shifting the membrane potential of the smooth muscle cell to a state of hyperpolarization. This, in turn, reduces the opening probability of Ca^2+^ channels involved in membrane depolarization and, thus, excitation is reduced [94, 95]. K^+^-channel openers are believed to hyperpolarize and reverse the tension of smooth muscle cells by a direct action on the cell membrane. During the last years, different types of K^+^-channels have been identified, some of them characterized by a specific distribution in the organs and tissues of animals and man [96].These research efforts have been paralleled by the development of various PCOs able to induce relaxation of both vascular and non-vascular smooth muscle. Drugs that inhibit smooth muscle activity by opening K^+^-channels have been considered as pharmacological tools for the treatment of cardiovascular and pulmonary diseases and dysfunctions of the urogenital tract [97–101]. In an in vitro study using the tissue bath technique, the effects were investigated of the PCOs levcromakalim (BRL 38227), rimakalim (HOE 234) and S 0121 on the contractile activity of isolated human ureteral smooth muscle induced either by KCl or transmural electrical field stimulation (EFS),. Both the tension of the tissue exerted by KCl and the phasic contractions induced by means of EFS were dose-dependently reversed by the drugs with different degrees of magnitude, with HOE 234 being more effective in reversing the tension induced by KCl. In the EFS experiments, only minor effects of the drugs were detected on the contraction amplitude of the tissue. HOE 234 also exerted significant relaxation of isolated human vascular tissue contracted by the alpha-adrenoceptor agonist norepinephrine [102]. de Moura & de Lemos-Neto (1996) in their study observed an attenuation induced by BRL 38227 of both the pre-synaptical phasic activity and also tonic contractions of isolated human distal ureter, these effects were inhibited by the K^+^-channel blocker glibenclamide [103]. In the anesthetized male rabbit, the effects were investigated of therapeutic concentrations of BRL 38277, HOE 234 and S 0121 on ureteral peristalsis (frequency, pressure amplitude, intraluminal pressure), as well as on blood pressure. BRL 38277 and S 0121 resulted in a minor reversion only of the phasic contractile activity of the ureter. The effects of the drugs on the cardiovascular system were more pronounced. HOE 234 induced a complete inhibition of the amplitude and frequency of ureteral peristalsis, this effect was accompanied by a dramatic drop in blood pressure [104]. Based on the findings up until today, it seems unlikely that a therapeutic dose of any of the PCOs exemplified above can be used to effectively treat non-complicated ureteral colics due to urinary stone disease and selectively abolish spasmic contractions of the ureter. Moreover, the use of some PCOs is strongly limited by the potent cardiovascular effects exerted by the compounds. However, further development of ureter-selective PCO agents not severely altering in vivo hemodynamics, such as cyanoguanidine analogues of cromakalim or the carbamic acid ethyl ester retigabine, may require additional research [105, 106].

## Concluding remarks

**Fig. 1:**
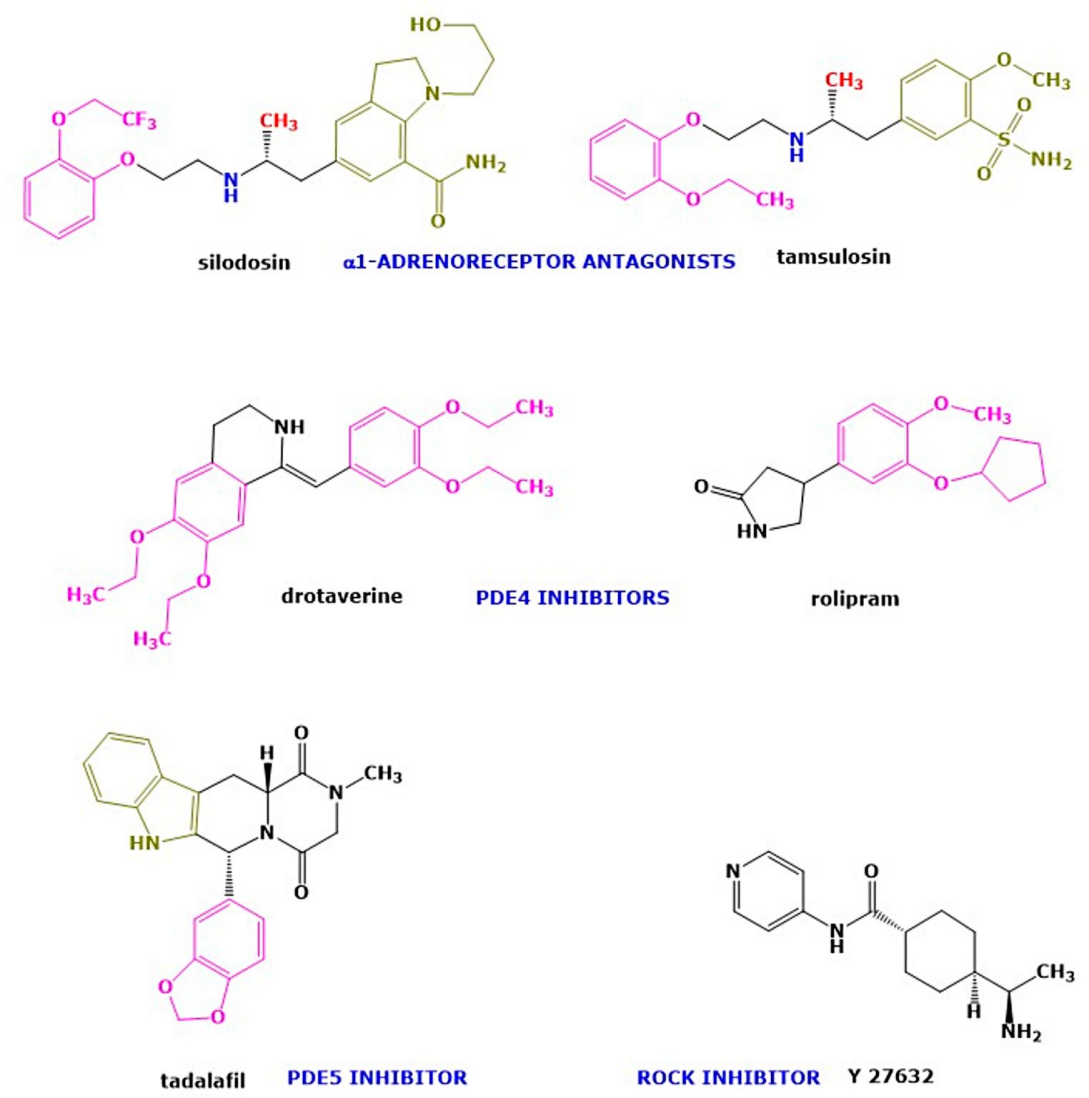
Structural formulas of some compounds assumed to gain clinical significance in the near future in the treatment of ureteral stone disease by facilitating relaxation of ureter smooth muscle and, thus, promoting the spontaneous expulsion of ureteral calculi and easing episodes of colic pain: alpha 1-adrenoceptor antagonists silodosin and tamsulosin, the PDE4 (cyclic AMP PDE) inhibitors drotaverine and rolipram, PDE5 (cyclic GMP PDE) inhibitor tadalafil (interacting with the signaling pathways mediated via cyclic AMP or NO/cyclic GMP, respectively) and the Rho kinase (ROCK) inhibitor Y 27632

Compared to other pharmacological principles for patients with ureter stones, many more studies have been conducted on the efficacy of alpha1-AR antagonists to facilitate stone expulsion. The use of alpha 1-AR antagonists to reduce basal ureter smooth muscle tonus in order to dislodge a stone that then may be propelled downwards the urinary bladder by peristaltic contractions is supported by some substantial data. However, translation from the lab into the clinic of the use of alpha-1-AR antagonists as MET has, in part, disclosed variable results. Since evidence for other pharmacological monotherapy principles in ureter stone disease is less robust, it seems that alpha 1-AR antagonists, depending on stone characteristics and location, may currently be the better option for MET in selected populations of patients [107]. Beta-AR-mediated regulatory functions of ureter smooth muscle tonus are interesting and relate to positive findings using the beta 3-AR-antagonist mirabegron. Nevertheless, additional information is of importance to assess and validate the preclinical and clinical pharmacology of this drug in ureter stone disease. Based on the putative relaxant role of signals mediated via cyclic AMP and cyclic GMP in the junction between the ureter and the urinary bladder, taken together with clinical MET information on agents targeting this pathway, inhibitors of the PDE4 (cyclic AMP PDE) and PDE5 (cyclic GMP PDE), such as rolipram drotaverine and tadalafil, seem to be an intriguing pharmacological approach to be extended in larger clinical trials [74, 75, 78, 108] (see Fig. 1). In addition, positive information on the efficacy yielded by combinations of currently available drugs, e.g. PDE5 inhibitors that facilitate relaxation by increasing cyclic GMP in ureter smooth muscle, and alpha 1-ARs antagonists that block ureter smooth muscle contraction, suggests beneficial effects on stone expulsion that might be superior to those brought about by monotherapies and should, thus, be investigated further. While NO donors, Ca^2+^ channel antagonists and K^+^-channel openers may be limited for further development for MET due to their systemic effects and a lack of effect on stone clearance, ROCK inhibitors would be an interesting option to be explored further as a future pharmacological principle in ureter stone disease Table 1.

**Table 1:** Clinical and experimental drugs currently considered for the use in human ureteral stone diseases (AR = adrenoreceptor, cyclic AMP = cyclic adenosine monophosphate, cyclic GMP = cyclic guanosine monophosphate, GTN = glycerol trinitrate, MET = medial expulsion therapy, MLC = myosin light chain, MR = muscarinic receptor, NO = nitric oxide, SNP = sodium nitroprusside, ISDN = isosorbide dinitrate

Class of drugs	Drugs	Pharmacological action
alpha 1-AR antagonists	silodosin, tamsulosin	inhibition of phospholipase C-dependent contractility
beta-AR agonists	salbutamol, mirabegron	activation of smooth muscle contractility/relaxation
MR antagonists	tolterodine	activation of relaxation
nitric oxide donors	SNP, SIN-1, ISDN, GTN	NO/cyclic GMP formation, relaxation
phosphodiesterase (PDE) inhibitors	drotaverine, rolipram, tadalafil, sildenafil, vardenafil	inhibition of PDE activity, maintenance of cyclic AMP and cyclic GMP levels
Rho kinase (ROCK) inhibitors	Y 27632 (not yet used in MET)	inhibition of Ca^2+^-independent MLC phosphatase activity
Ca^2+^ channel antagonists	nifedipine (little effective)	inhibition of Ca^2+^-dependent contraction
K^+^ channel openers	cromakalim, retigabine	potentially useful to decrease smooth muscle tension

## Data Availability

No original datasets were generated or analysed during the current study.
